# Multi-origin mucinous neoplasm: Should we prophylactically remove the appendix in the setting of mucinous ovarian tumors?

**DOI:** 10.1016/j.ijscr.2019.12.027

**Published:** 2019-12-26

**Authors:** Misbah Yehya, Matthew Denson, Zbigniew Moszczynski

**Affiliations:** Department of Surgery, Carepoint Health Bayonne Medical Center, Bayonne, NJ, USA

**Keywords:** Appendiceal tumors, LAMN, Mucinous neoplasm, Multiple primaries, Ovarian tumors, Prophylactic appendectomy

## Abstract

**Introduction:**

Tumors of the ovary and appendix have been well documented in the setting of pseudomyxoma peritonei (PMP) with constant debate over tumor origin. Generally, these tumors are found to have a single primary origin, most commonly the appendix, with metastatic spread to the ovaries.

**Care presentation:**

Here we present a 61-year-old female who underwent total abdominal hysterectomy and bilateral salpingo-oophorectomy (TAH-BSO) for a primary mucinous ovarian carcinoma. She presented to our institution one year later with abdominal pain and a palpable right lower quadrant mass, which on histopathologic exam was found to be a primary low grade mucinous appendiceal neoplasm (LAMN), alluding to the potential of two separate primary disease processes.

**Discussion/conclusion:**

With two primary, non-synchronous lesions, a thorough literature review suggests that during the patient's initial TAH-BSO, she could have additionally undergone an appendectomy. In doing so, this would provide accurate, complete staging and determine if the two neoplasms were truly primary in origin or metastatic. In addition, new genetic markers are being discovered, such as the Special AT-rich sequence-binding protein 2 (SATB2) marker, which has been found to be positive in those with a LAMN and negative in those with a primary mucinous ovarian carcinoma. By acquiring appropriate and complete staging we can better diagnose and treat these neoplasms.

## 1 Introduction

Tumors of the ovary and appendix have been well documented in the setting of pseudomyxoma peritonei (PMP) with constant debate over tumor origin. Generally, these tumors are found to have a *single* primary origin, most commonly the appendix, with metastatic spread to the ovaries.

In this report, we discuss a patient who presented to the emergency room with right lower quadrant abdominal pain and a palpable mass for 2 months. Her past medical and surgical history included primary mucinous ovarian carcinoma with TAH-BSO and no chemotherapy/radiation. A computed tomography (CT) scan of the abdomen and pelvis demonstrated a 7.6 × 4 cm mass with peripheral calcifications suspicious for a mucocele versus malignancy. The patient underwent surgical exploration with excision of the mass that was well tolerated. Histopathology confirmed a primary low-grade appendiceal mucinous neoplasm.

There are few documented cases of synchronous primary ovarian and appendiceal mucinous neoplasms; however, there have been very few recorded cases of two non-synchronous primary neoplasms and their appropriate diagnosis and treatment prompting this case report and literature review. Our work has been reported in line with the SCARE criteria [9].

## 2 Case presentation

**Fig. 1:**
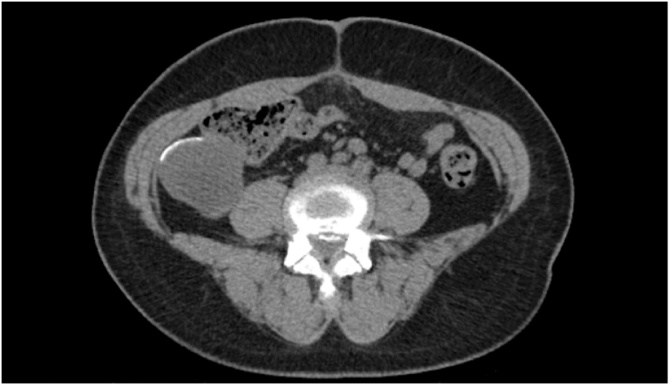
Computed tomography of the abdomen and pelvis demonstrating a 7.6 × 4 cm mass with peripheral calcification suspicious for a mucocele versus mucoid epidermoid carcinoma.

Our patient is a 61-year-old Jamaican female who recently immigrated to the United States, with a past medical history significant for mucinous ovarian carcinoma status post TAH-BSO in Jamaica, 2018. The patient did not undergo any post-operative chemotherapy due to the low malignant potential of her initial pathology. Approximately one year later, she presented to our emergency department with right sided abdominal discomfort for the past two months. The rest of patient’s history was unremarkable. There were no other accompanying symptoms, namely change in bowel habits or weight loss. On physical exam, the patient had a palpable mass on the right side of her abdomen that was tender on palpation. A CT scan of the abdomen and pelvis demonstrated a 7.6 × 4 cm mass with peripheral calcification suspicious for a mucocele versus mucoid epidermoid carcinoma [Fig. 1]. Subsequent pelvic and abdominal magnetic resonance imaging also demonstrated findings suspicious for a mass abutting the cecum concerning for an appendiceal mucocele. Additionally, the cancer antigen 125-5 was found to be elevated at 49.6 U/mL.

**Fig. 2 and 3:**
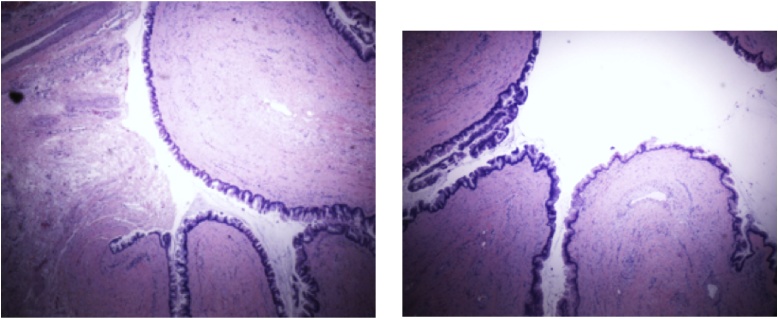
Histopathologic slides of the omentum and appendix demonstrating a low grade appendiceal mucinous neoplasm (LAMN). Histochemical analysis revealed CK7- and CK 20+ markers, confirming appendiceal origin or the tumor.

With a presumed diagnosis of appendiceal mucocele versus malignancy, the patient agreed to surgical intervention. The patient was taken to the operating room for an exploratory laparotomy. Upon inspection of the abdominal cavity, the patient was found to have a mass in the right lower quadrant which appeared to be originating from the appendix. Numerous omental implants were identified warranting an omentectomy, as well as several deep pelvic gelatinous deposits which were removed with sharp dissection. The right lower quadrant mass appeared intact, freely mobile, and limited to the distal tip of the appendix. There was limited surrounding inflammation, with mild fibrous adherence to the right paracolic gutter. After careful dissection, the appendix with the associated distal mass were removed and sent for pathology.

### 2.1 Cyto-histopathology results

The omentum was found to have a focus of low-grade mucinous neoplasm consistent with an appendiceal origin. The appendix was found to be a low grade appendiceal mucinous neoplasm with peritoneal involvement [Figs. 2 and 3]. The proximal end of the appendix was free of neoplasm. The pelvic implants were also noted to have foci of low-grade mucinous neoplasm consistent with an appendiceal origin. Immunohistochemical staining of ovarian primary were completely absent.

The patient had an uneventful postoperative course and recovered without complications. She was discharged on postoperative day three with continued follow up in our clinic.

## 3 Discussion

Although a distended, mucus-filled appendix is often called a mucocele, this term is ambiguous and best used to describe a radiological finding rather than a pathologic entity. In 2012, the Peritoneal Surface Oncology Group International (PSOGI) developed a consensus classification that has helped to resolve much of the confusion surrounding diagnostic terminology [2]. In the realm of non-neoplastic versus neoplastic mucinous lesions, our presenting case was classified as a low-grade appendiceal mucinous neoplasm (LAMN).

These lesions are rare, with only one to two thousand cases diagnosed annually in the United States [1]. Whether benign or malignant, there is a slight predominance towards females. Laboratory findings are generally nonspecific, but patients may present with elevated tumor markers including CEA, CA 19-9, and/or CA 125-5.

The LAMN is defined as a true neoplasm with abundant mucin production as well as dysplastic epithelium. Despite their bland appearance, LAMNs may penetrate through the appendiceal wall, cause appendiceal rupture, and progress to pseudomyxoma peritonei (PMP) [1]. Staging can further help delineate treatment protocol and outcome management. In our case, the patient’s appendiceal neoplasm was stage T4a, invading through the visceral peritoneum involving the serosa as well as M1b designating isolated intraperitoneal metastasis. Our case’s cyto-histopathology confirmed the mass and its associated omental and pelvic samples were of appendiceal origin (CK7- and CK20+).

There has long been debate of which tumor, ovarian or appendiceal, is the site of origin in the setting of pseudomyxoma peritonei (PMP). PMP is a rare disease in which the mucin cells are distributed within the peritoneal cavity. Although most literature states PMP’s origin is appendiceal in nature, many other tumors including ovarian, stomach, and pancreas have been documented. These mucinous cells continue to proliferate which eventually causes the signs and symptoms seen during patient presentation. The tumors continue to grow and can perforate, causing seeding with the peritoneal cavity and leading to possible metastasis.

This patient was found to have a primary mucinous ovarian carcinoma in early 2018 diagnosed in her home country of Jamaica. In March of 2019, once she immigrated to the United States, she was found to have a primary mucinous appendiceal neoplasm (LAMN) with cyto-histopathology confirming no primary ovarian lesion. In the available literature, synchronous tumors of the ovary and appendix are an uncommon yet well-recognized occurrence in the setting of PMP [8]. The origin of such synchronous tumors is widely debated with most evidence favoring a primary appendiceal tumor with the ovarian tumor representing a metastatic process. In our case, the patient was diagnosed with separate primary ovarian and appendiceal mucinous neoplasms almost a year apart. Further investigation is needed to determine the risks of being diagnosed with more than one primary mucinous tumor over time. Although there is no data supporting a genetic component to this disease, patients with multiple primary mucinous tumors may have a possible genetic preponderance that is not currently established.

In 2018, our patient was diagnosed with the mucinous ovarian carcinoma and underwent a (TAH-BSO) with no further treatment thereafter. A recent 2017 study conducted in Denmark, published in the International Journal of Gynecological Cancer, aimed to assess the importance of an appendectomy in the presence of a mucinous ovarian adenocarcinoma as it can be difficult to distinguish between primary ovarian and primary appendiceal cancers clinically, histologically, and immunohistochemically [3]. Essentially, the appendix is needed for complete and thorough staging of a presenting tumor. As such, incomplete staging can affect overall prognosis and patient outcome. The study concluded that failure to perform an appendectomy correlated with a worse prognosis. A normal-looking appendix does not exclude metastatic disease, and because an appendectomy is easily performed and does not increase morbidity, it should be performed during surgery for suspected mucinous ovarian cancer [3]. Many studies have been conducted to ascertain whether an appendectomy is beneficial in the setting of metastatic disease with some studies against appendectomies if the appendix is visually normal [7]; however, our case is complicated because both neoplasms were independent, primary tumors presenting a year apart. The question remains, should the patient have had an appendectomy at the time of her TAH-BSO? Further studies would need to be conducted to determine the benefit of an appendectomy for all patients diagnosed with a primary mucinous ovarian neoplasm and its correlation and disease potential with non-metastatic, primary appendiceal mucinous neoplasms.

There is an extensive immunohistochemical panel that assesses expressions of various markers; however, even then, differentiation is difficult and pathologist dependent. Our patient’s current immunohistochemical analysis tested for CK7 and CK20 which help differentiate an appendiceal versus ovarian mucinous neoplasm. With recent advancements in gene sequencing, the March 2018 issue of Pathology Research and Practice published an article with evidence of a new supportive marker for the differentiation of a primary mucinous tumor of the ovary and an ovarian metastasis of a low-grade appendiceal mucinous neoplasm (LAMN), the Special AT-rich sequence-binding protein 2 (SATB2) marker. The SATB2 expression is primarily within the gastrointestinal tract. Patients with physical, radiographic and/or pathologic evidence of an ovarian mass with concomitant LAMN, in the context of pseudomyxoma peritonei (PMP) or small foci of peritoneal spread, the addition of the SATB2 marker in immunohistochemical staining revealed strong expression of the marker in those with LAMN while SATB2 was negative in cases that demonstrated cyto-histopathologic primary mucinous ovarian carcinomas [4]. Our patient was not assessed for SATB2 expression of either her ovarian or appendiceal neoplasms. Further analysis could help differentiate the two neoplasms as truly primary or possible metastatic potential that would not be seen with the typical immunochemistry analysis performed on such specimens.

## 4 Conclusion

The presented case demonstrates a rare variant in mucinous neoplasms: a primary ovarian and primary appendiceal neoplasm diagnosed and treated approximately one year apart in two different countries. Although there is no literature to support a genetic anomaly that increases the risk of mucinous tumors throughout the body, with gene sequencing and newly developing biotechnology, there may be a biologic component not currently recognized. In addition, diagnosing mucinous neoplasms based on pathology is challenging even amongst seasoned pathologists. More detailed immunological studies with more than one pathologist overlooking the case may be warranted as new markers, including the SATB2, continue to be discovered. Furthermore, adequate staging is a necessity because of this disease diagnostic challenges and routine appendectomy in the setting of primary ovarian mucinous neoplasms may be warranted to achieve the proper diagnosis and treatment for patients. These new markers and staging protocols can continue to help us accurately diagnose primary versus metastatic disease processes and achieve the best outcomes for our patients in the future.

In our case, with stage T4a LAMN, regularly scheduled imaging and tumor marker follow up can permit earlier detection of recurrent disease, which occurs between 4.8 and 20.4 % of the time [1]. Current recommendations vary from imaging every six months to two years [5,6].

## Sources of funding

There were no study sponsors involved in the submitted work.

## Ethical approval

This is not applicable to the submitted work.

## Consent

Written informed consent was obtained from the patient for publication of this case report and accompanying images.

## Authors contribution

Study Concept/Design – Misbah Yehya, Matthew Denson, Zbigniew Moszczynski.

Data Collection - Misbah Yehya, Matthew Denson, Zbigniew Moszczynski.

Writing the Paper – Misbah Yehya.

## Registration of research studies

This is not applicable to our submitted work.

## Guarantor

The Guarantor’s are Misbah Yehya, Matthew Denson, and Zbigniew Moszczynski.

## Provenance and peer review

Not commissioned, externally peer-reviewed.

## Declaration of Competing Interest

There are no conflicts of interest that influence the submitted work.
